# Data of a PV plant implemented in hot semi-arid climate

**DOI:** 10.1016/j.dib.2021.106756

**Published:** 2021-01-14

**Authors:** Nour-eddine Id Omar, Lahcen Boukhattem, Fahd Oudrhiri Hassani, Amin Bennouna, Aziz Oukennou

**Affiliations:** a LMPEQ, National School of Applied Sciences, Cadi Ayyad University, 40000 Marrakech, Morocco; b EnR2E laboratory, National Center for Studies and Research on Water and Energy (CNEREE), Cadi Ayyad University, 40000 Marrakech, Morocco; c Laboratory of Nanomaterials for Energy and Environment (LN2E), Physics Department Faculty of Sciences Semlalia, Cadi Ayyad University 40000 Marrakech, Morocco; d Advanced Control of Electrical Systems Team - LESE, ENSEM, Hassan II University, 8118 Casablanca, Morocco

**Keywords:** PV power generation, Weather data, PV module temperature, Hot semi-arid climate, PV power forecast

## Abstract

This paper presents data collected from a 5.94 kWp grid connected photovoltaic (PV) plant implemented in hot semi-arid climate of Safi region, Morocco. The data include electrical power production and PV module temperature of three PV technologies: mono-crystalline (m-Si), poly-crystalline (p-Si), and amorphous (a-Si); they also include plane of array solar irradiance and ambient temperature. Solar irradiance was measured with calibrated reference cells, inverters provided the produced powers, and the temperatures were obtained by Pt100 probes. The data were measured each 5 min and were remotely accessible through internet. They were preprocessed to eliminate unrepresentative records and were used for the development of simple and accurate models for PV power forecasting [1]. These data are typical for hot semi-arid climate and may be reused for regional forecast of PV power as well as solar energy and PV module temperature predictions.

## Specifications Table

**Table:** 

Subject area	Renewable Energy, Sustainability and the Environment.
More specific subject area	Solar energy, photovoltaics, forecasting models.
Type of data	Excel files.
How data was acquired	Plane of array solar irradiance and ambient temperature were respectively measured by a calibrated poly-crystalline reference cell and Pt100 temperature sensor. Electrical data were gotten from inverters. Surface temperatures were measured with probes installed on the rear side of the PV modules.
Data format	Raw, filtered, preprocessed.
Parameters for data collection	The 5.94 kWp grid connected PV plant is tilted by 32° and south oriented. It was implemented in hot semi-arid climate on the rooftop of a public institution without a regular cleaning process.
Description of data collection	All the data were recorded with 5 min time step. Calibrated reference cell, Pt100 sensors and inverters are connected with a data acquisition system which stores the data in daily Excel files and they were remotely accessible through internet.
Data source location	*Institution:* National School of Applied Sciences *City:* Safi *Country:* Morocco *Latitude and longitude* (32.3265°N; -9.2634°W)
Data accessibility	*Repository name:* Data of three silicon-based PV technologies implemented in hot semi-arid climate. *Data identification number:*http://dx.doi.org/10.17632/b2s4ycc2gz.1 *Direct URL to data:* http://dx.doi.org/10.17632/b2s4ycc2gz.1
Related research article	N. Id omar, L. Boukhattem, F. Oudrhiri Hassani, A. Bennouna, A. Oukennou, Power forecasting of three silicon-based PV technologies using actual field measurements, Sustain. Energy Technol. Assessments. 43 (2021) 1–15. https://doi.org/10.1016/j.seta.2020.100915.

## Value of the Data

• These data are representative for other sites characterized by hot semi-arid climate, i.e., the same measured weather data will be obtained and subsequently identical electrical and PV module temperatures data will be gotten for similar PV technologies. These data may also be used to get an overall sight on the PV modules behavior.
• The data are useful for the research community, policymakers, individuals and organizations interested in PV/solar power applications.
• They can be reused to develop new PV power forecasting models for short or long times horizon. They might also be combined with other data in different sites to perform a regional forecast of PV production or solar power availability.
• A combination of the measured weather data with the sun position and geographical coordinates can be used to develop solar power prediction methods.
• The provided data may be used for PV system performance comparison in different climates and under various operating conditions of module temperature and solar irradiance.
• The data may be useful for temperature prediction models of the PV modules in different locations characterized by hot semi-arid climate.

## 1 Data Description

The collected raw data refer to a 5.94 kWp grid connected PV plant implemented on the rooftop of National School of Applied Sciences of Safi, Morocco. This location is characterized by hot semi-arid climate [2]. The raw data include:

- The most important weather variables: global horizontal irradiance, plane of array irradiance, and ambient temperature;
- The most important electrical variables of each PV technology obtained from inverters: DC power, DC current and voltage, AC power, AC current and voltage, instantaneous and cumulative produced energy, grid injection frequency, operating and feed in time, inverter operating temperature;
- Module temperature of each PV technology (Tc).

All these features were measured 24 hours a day with a time step of 5 min. They were provided in daily separated csv files stored on SD cards and were received by email each day at midnight. On a single day, 7 separated csv files were received and named according to the day's date measurements. For instance, Table 1 illustrates the received csv files on June 9, 2018.

**Table 1:** Received csv files on June 9, 2018.

Data	PV technology	File name
Weather data	-	Safi-meteo20180609
Electrical data	m-Si	Safi-MO-Spot-20180609
	p-Si	Safi-PO-Spot-20180609
	a-Si	Safi-AM-Spot-20180609
Module temperature	m-Si	Safi-Tc-Mono20180609
	p-Si	Safi-Tc-Poly20180609
	a-Si	Safi-Tc-Amorphe20180609

All collected raw data are provided in a folder named ‘Raw Data’. The latter comprises seven excel files which contain all the merged daily weather data files; daily electrical data files of m-Si, p-Si and a-Si technologies, and their corresponding daily PV module temperatures files. These excel files are named respectively ‘Weather data’, ‘Electric m-Si’, ‘Electric p-Si’, ‘Electric a-Si’, ‘Tc m-Si’, ‘Tc p-Si’, and ‘Tc a-Si’.

These raw data contain some gaps due to [3]:

- Electrical breakdowns: the PV plant's outputs are synchronized with the same frequency as that of the grid; when the grid power cuts-off, the data acquisition system doesn't work;
- Inverters failure: when an inverter is faulty, electrical records are set to 0 until it is fixed;
- Burned or disarmed fuse: in these cases, null or erroneous values are stored.

Despite the presence of some missing periods, the provided data can be successfully reused as mentioned previously in the Value of the data section. However, in the case of time series models where continuously measured data are usually required, an appropriate estimation method (average/weighting methods [3], Numerical Weather Predictions, prediction models…) can be used to fill the gaps due to the aforementioned causes.

The provided raw data were preprocessed to eliminate outliers and were then used for PV power forecasting [1]. The preprocessed data include DC power productions (P_m-Si_, P_p-Si_ and P_a-Si_) and module temperatures (T_c_m-Si_, T_c_p-Si_ and T_c_a-Si_) of the three PV technologies. They also include weather features highly affecting the PV modules behavior: plane of array solar irradiance (POAI) and ambient temperature (Ta). The preprocessed data of the three PV technologies were gathered together on separated sheets of an Excel file named ‘Preprocessed Data’. The data of m-Si and a-Si technologies were both spread over a period from June 18, 2016 to July 15, 2018. The p-Si technology data were extended over a period from May 25 to December 30, 2018.

The different steps adopted during the data preprocessing technique are further described in the next section.

## 2 Experimental Design, Materials and Methods

### 2.1 PV plant description

The PV plant (Fig. 1 provided in [1]) was installed in the framework of the well documented ‘Propre.Ma’ national project [3]. It is composed of three PV technologies: mono-crystalline (m-Si), poly-crystalline (p-Si) and amorphous (a-Si); accumulating an overall capacity of 5.94 kWp. Both m-Si and p-Si subarrays were built with eight PV modules of 255 kWp each connected in series, whereas the a-Si subarray is constituted of two strings of 6 modules with 155 kWp each. The PV modules were mounted on a metallic structure tilted by 32° and south oriented. Electricity produced by the PV plant is fed into the grid through three identical inverters. Further technical characteristics of the installed PV plant are summarized in Table 2 given in [1].

The main climatic variables highly affecting the PV plant's production were measured by a weather station installed nearby the PV array. The recorded features include global solar irradiance on the tilted PV modules surface measured with calibrated crystalline silicon reference cell with calibration uncertainty less than 2.3 %, and ambient temperature collected with Pt100 sensor with accuracy better than 0.1 °C [4]. Besides, PV module temperatures were measured on the backside of each technology using Pt100 sensors, with accuracy better than 0.1 °C [4]. DC powers were also obtained directly from the inverters equipped with maximum power point tracking function.

All aforementioned data were recorded simultaneously during each time step of 5 min and stored in daily files which were remotely accessible through internet [4].

### 2.2 Pre-processing data

The above described raw data contain some gaps due to dis-functioning of the monitoring system. For each technology, a single file containing weather, electrical and PV module temperature data was built by following the steps hereafter:

1. Separated daily files for weather, electrical and module temperature data were gathered, each, in a single file. Furthermore, unnecessary variables were removed for both weather and electrical data. Only plane of array irradiance, ambient temperature and instantaneous produced DC power were kept;
2. The three files (weather, electrical and module temperature data) were regrouped in a single file so that the measurement's time of the data is exactly the same, i.e., at a point in time, weather data, PV power production and module temperature are all in the same line of the new csv file;
3. For electrical data, a little time delay might occur compared to weather data. To correct this temporal difference, PV power and solar irradiance were plotted in the same graph and a little translation of PV power's curve was done so that both of the curves coincide;
4. Incorrect measured observations due to equipment failure were excluded from the obtained new file;
5. Only data during daylight period were kept;
6. PV array efficiency $\eta_{PV\;array,t}$ (the amount of plane of array irradiance POAI converted by the whole PV subarray into electricity $P_{DC,\;measured}$) was assessed every 5 minutes using Eq. 1. Observations for which PV array efficiency is very high than module efficiency at STC conditions are excluded.
  (1) $$\eta_{PV\;array,\;t}=P_{DC,\;measured}\;/\;POAI$$
7. Apparent wrong measurements (negative values of features) were deleted from datasets. Then, each feature was treated independently using the box-plot method to eliminate extreme values; observations beside the interval of [Lower Extremity (LE), Upper Extremity (UE)] were dropped. LE and UE are defined by the following equations:
  (2) LE=Firstquartile−1.5×Interquartilerange
  (3) UE=Thirdquartile+1.5×Interquartilerange
8. Finally, PV power production was predicted by a linear model and influential points considered as outliers were identified by standardized residuals $e_{i}$ method, i.e., observations for which $|e_{i}|>2$ were removed.

## Credit Author Statement

**N. Id omar and L. Boukhattem:** Data collection, Data preprocessing, Writing the original version, Writing - review & editing; **F. Oudrhiri hassani:** Data collection, Writing the original version, Review; **A. Bennouna:** Review; **A. Oukennou:** Data collection.

## Declaration of Competing Interest

The authors declare that they have no known competing financial interests or personal relationships which have or could be perceived to have influenced the work reported in this article.
